# Supplementary material for: Rapid micro-immunohistochemistry
Source: Microsyst Nanoeng. 2020 Oct 19;6:94. doi: 10.1038/s41378-020-00205-2 (PMC8433409; doi:10.1038/s41378-020-00205-2)
Supplement: Supplementary file 3 — Supplementary Information [file 41378_2020_205_MOESM3_ESM.docx]

# Supplementary information

**Rapid micro-immunohistochemistry**

Robert D. Lovchik^1^, David Taylor^1,2^, Govind Kaigala^1*^

^1^ IBM Research Europe, Saeumerstrasse 4, CH-8803 Rueschlikon, Switzerland

^2^ Current address: Eidgenössische Technische Hochschule Zürich, Department of Mechanical and Process Engineering,

Sonneggstrasse 3, 8092 Zurich, Switzerland

# *gov@zurich.ibm.com

**SI.1** **Fabrication of microfluidic probe heads**

**
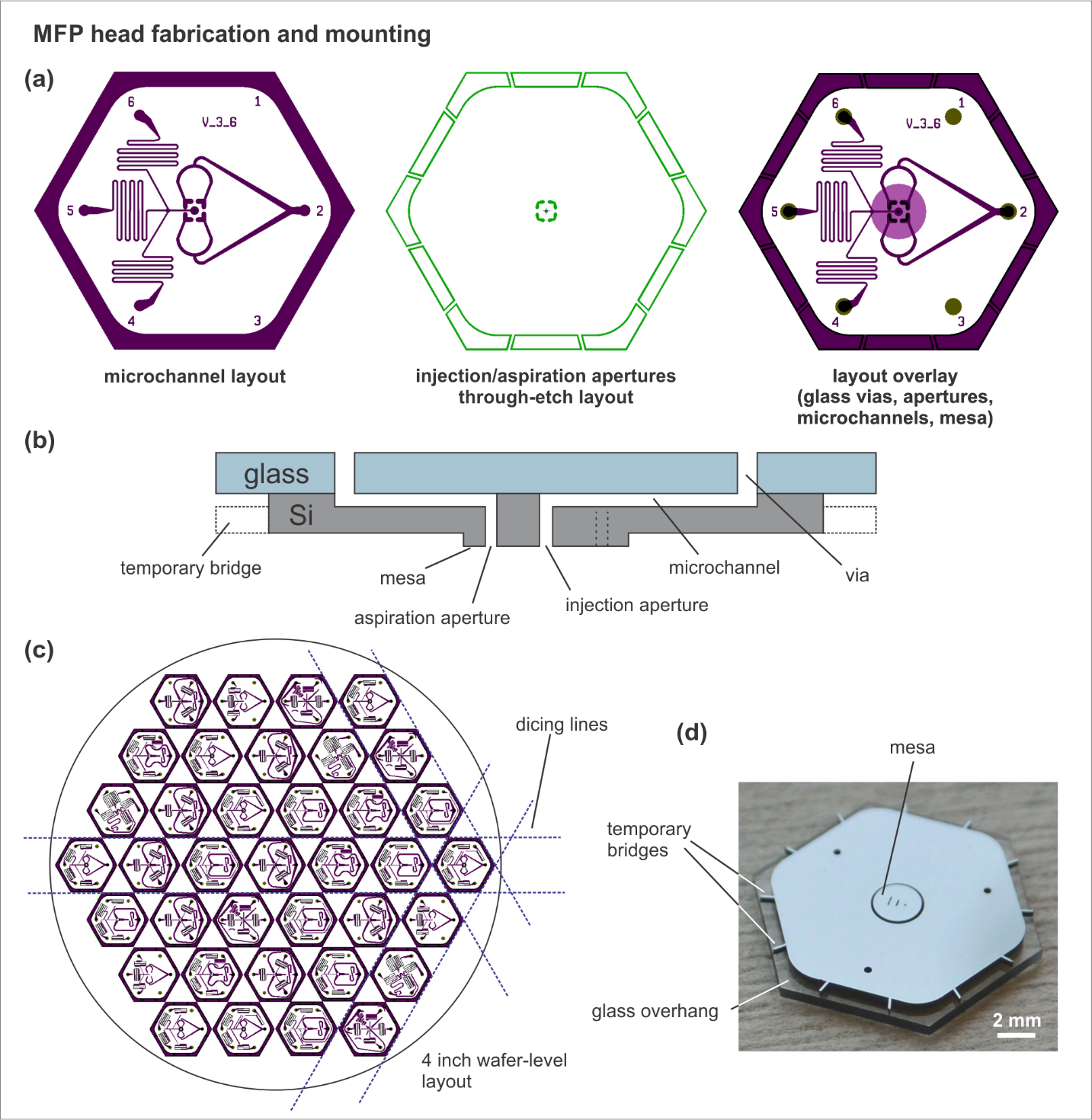
**

***Figure SI. 1.*** *Microfluidic probe heads and the fabrication steps. a) and b) The fluidic routing and the through-holes forming the apertures are implemented on opposing sides of a double-side polished Si-substrate. Briefly, the fabrication process involves etching and closing of the channels through anodic bonding to a glass substrate comprising vias for fluidic connection. c) Wafer layout with dicing-lines. d) Photograph of a horizontal probe head. The temporary silicon bridges are required to keep each probe head in place during the processing of the Si-wafer. The bridges are manually removed before a probe head is inserted into the holder.*

**SI.2** **Microfluidic probe platform**

**
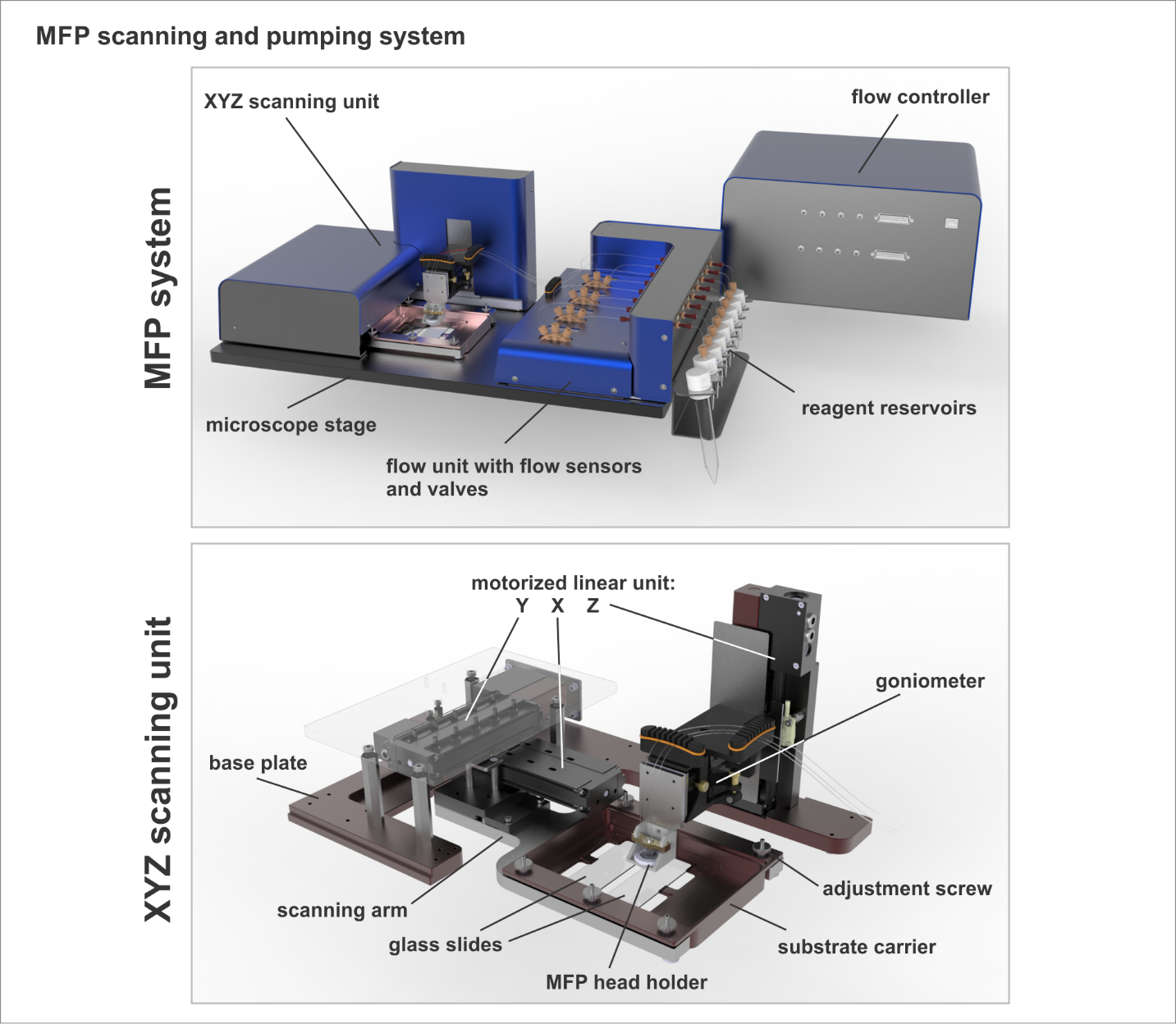
**

***Figure SI.2.*** *The microfluidic probe platform is composed of a custom-built XYZ scanning unit that is placed on the stage of an inverted microscope. The MFP head holder is linked to a goniometer mounted to the Z stage for alignment of the head relative to the substrate. The substrate is placed onto a substrate carrier that sits on the scanning arm. A pressure-driven pumping system is used to control the flow of liquids.*

**SI.3** **MFP head holder and associated components**

**
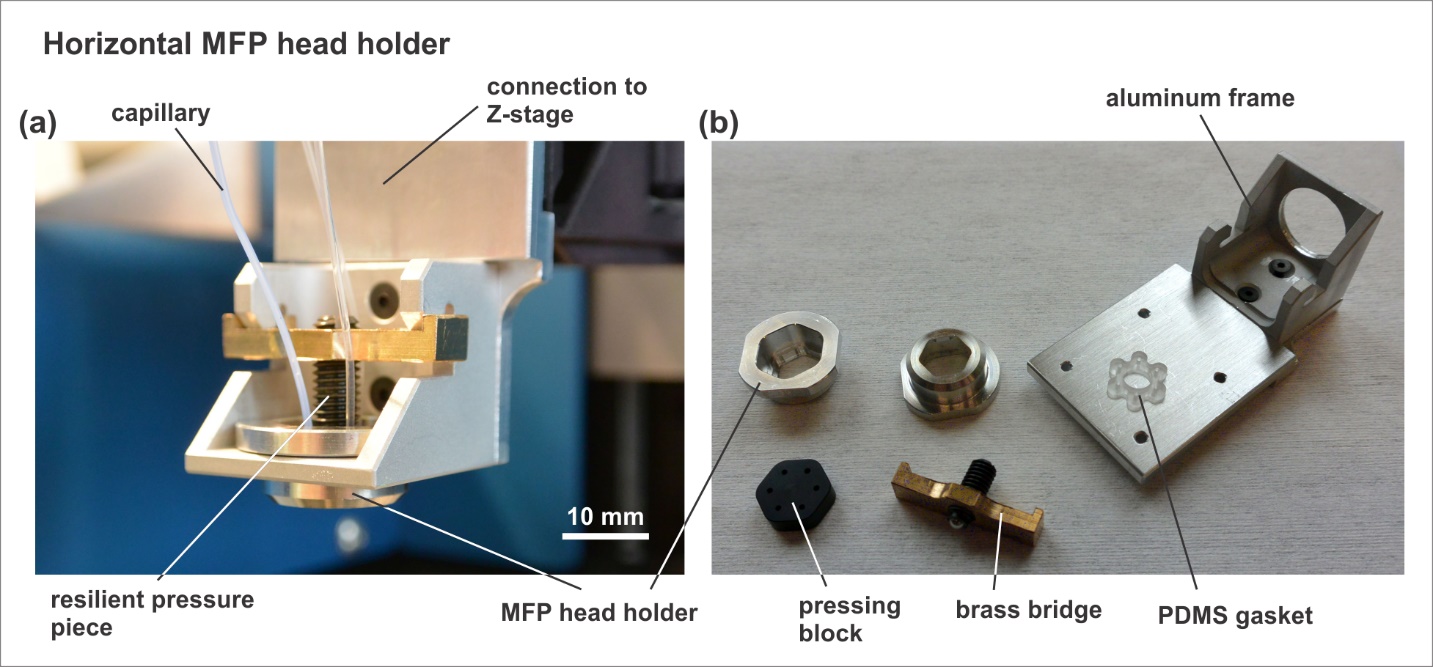
**

***Figure SI.3.*** *Photographs of the MFP head holder. (a) shows the zoomed-in view of a head within the holder, and (b) is an overview of all the individual components assembled to form the holder.*

**SI.4** **Dewaxing and target retrieval protocol**

**
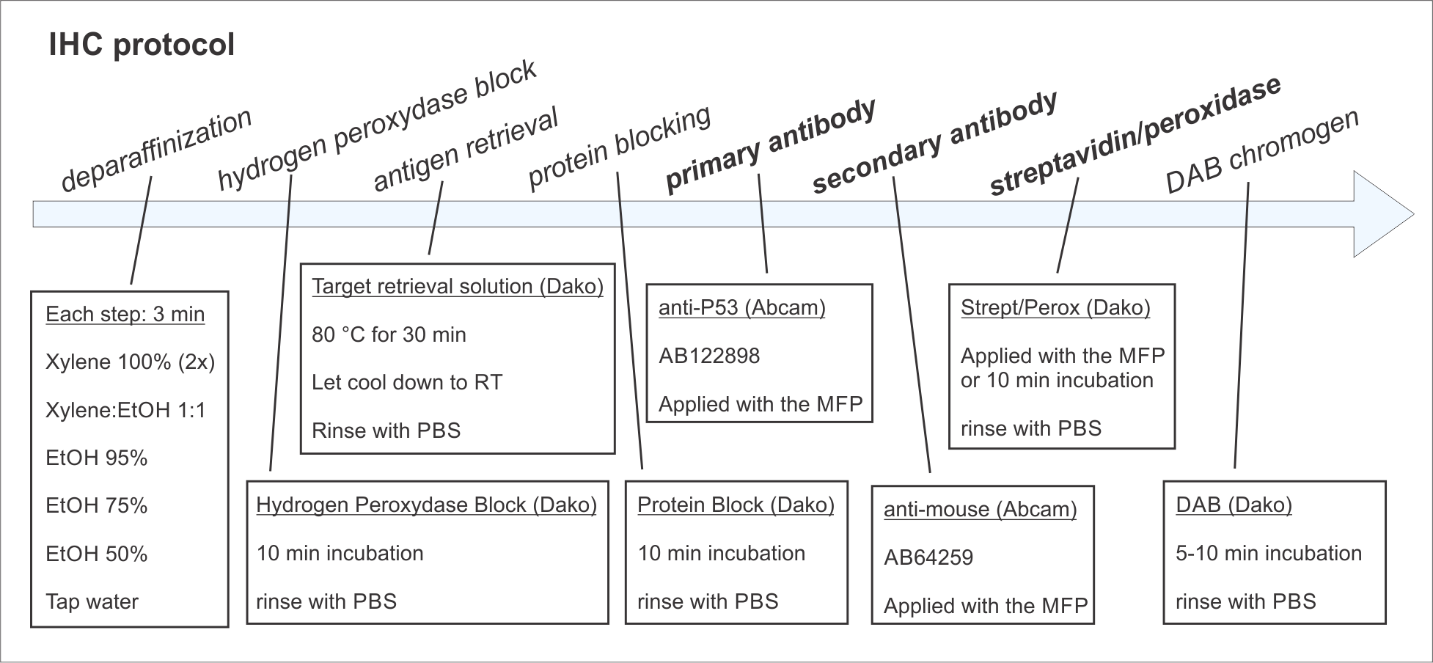
**

***Figure SI.4.*** *Sequence of steps for IHC-based staining with a focus on specific steps performed with the MFP. Prior to performing staining on a tissue section, it is important to remove the paraffin from the section and rehydrate the tissue. Depending on the sample being analyzed and the marker used, an antigen retrieval step may be performed. The subsequent steps of presenting the primary antibody, the secondary antibody and the color reaction are performed using the microfluidic chip.*

**SI.5 Incubation times**


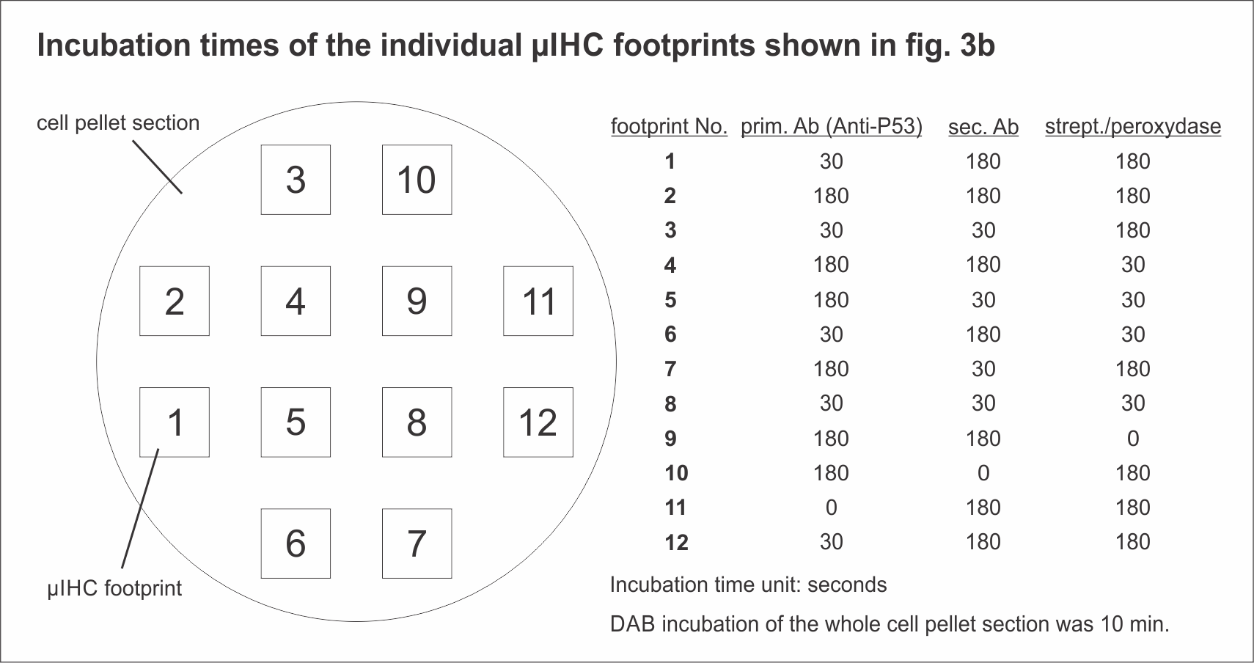


***Figure SI.5.*** *Location and corresponding incubation times of the µIHC footprints displayed in figure 3b.*

**SI.6 ANOVA table for discussed design of experiments**


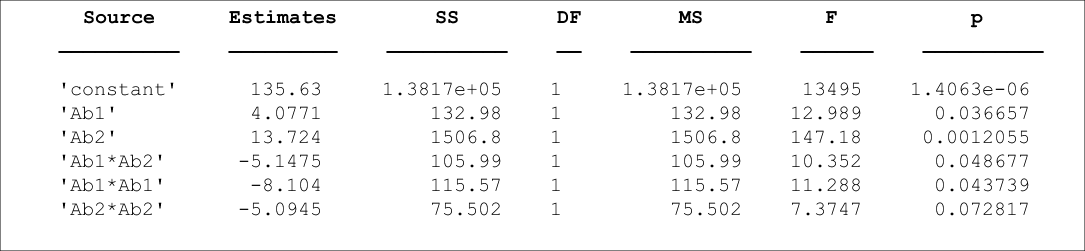


***Figure SI.6.*** *An effect is considered statistically significant if p<0.05, which is the case for linear and quadratic contributions of the primary antibody, linear contributions of the secondary antibody and the interaction between primary and secondary antibody.*

**SI.7 Photographs of anti-P53 stainings on cell block sections**
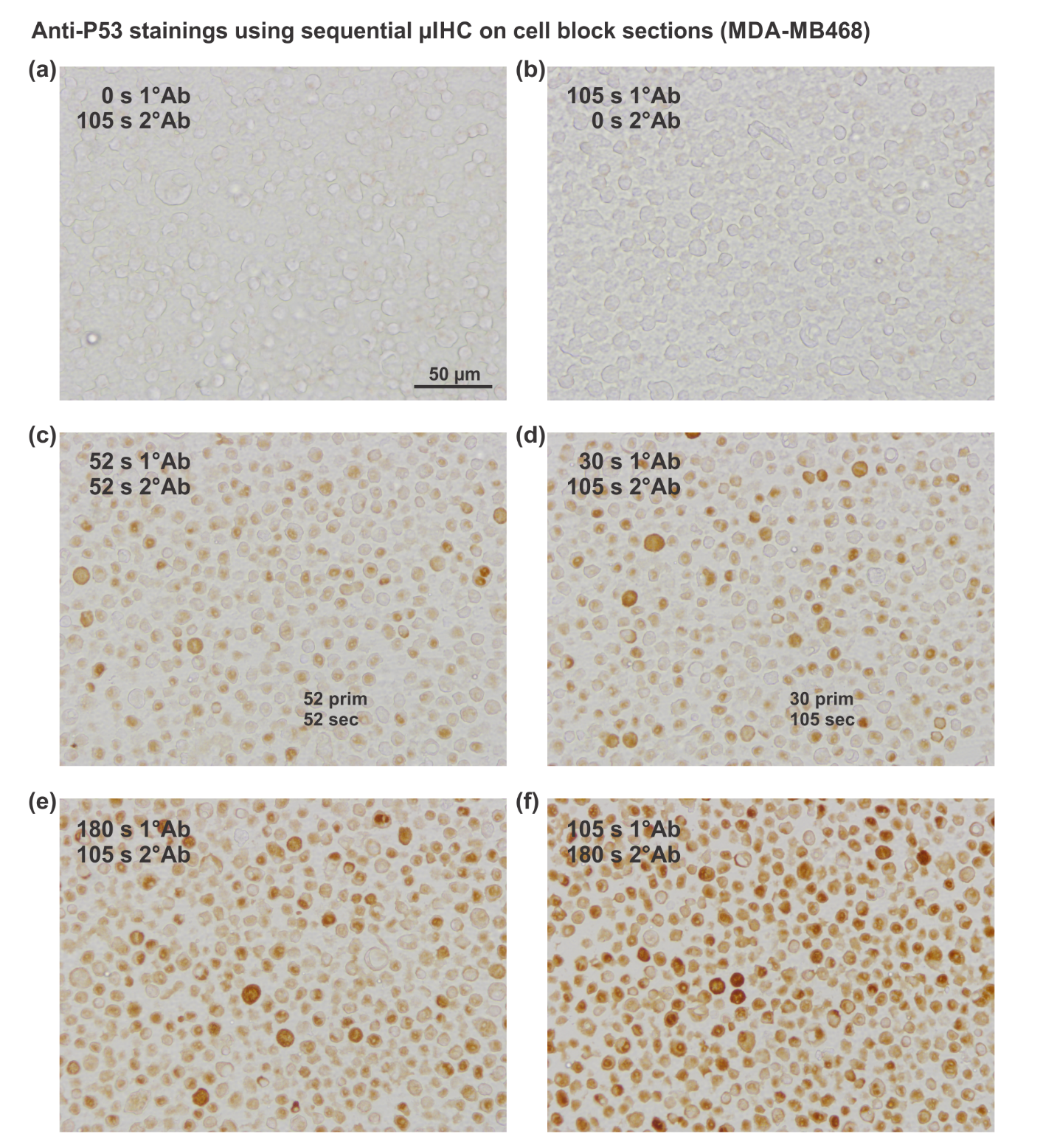


***Figure SI.7.*** *Staining results with anti-P53 on MDA-MB468 cell block sections using sequential chemistry applied by an MFP. (a) and (b) show negative controls where either the primary antibody or the secondary antibody were not applied. (c) to (f) show the staining results with different incubation times of the primary and secondary antibody.*
