# Supplementary material for: Rapid micro-immunohistochemistry
Source: Microsyst Nanoeng. 2020 Oct 19;6:94. doi: 10.1038/s41378-020-00205-2 (PMC8433409; doi:10.1038/s41378-020-00205-2)
Supplement: Supplementary file 4 — Supplementary Information [file 41378_2020_205_MOESM4_ESM.docx]

# *Microsystems & Nanoengineering*

Microfluidics: Rapid immunohistochemistry

A new microfluidic device enables immunohistochemistry analysis of tissue within minutes. Immunohistochemistry is a quantitative approach for the analysis of antigens in tissue sections, such as for the detection of cancer. Antibodies are introduced to the tissue that bind to specific antigens, enabling their detection. However, speeding up the diagnosis process, or detecting multiple antigens at one time, is challenging due to the complexity of antibody reactions with antigens. Here, a team from IBM Research Europe report an improved microfluidic process for immunohistochemistry. By using a horizontally oriented microfluidic probe they are able to speed-up reaction rates for the assay and switch between different processing liquids. This allows them to reduce the main incubation step time from an hour to less than 30 minutes.

Related article manuscript number: MICRONANO-01166R1

Article title: Rapid micro-immunohistochemistry

Corresponding author and affiliation/s: Govind V. Kaigala, IBM Research Europe, Rueschlikon, Switzerland

**About your Editorial Summary — please read**

**Before approving this Editorial Summary, please carefully check that (1) the summary text lists the correct author(s) and (2) the spelling and order of all author names and affiliations are correct.**

This **Editorial Summary** is based on your manuscript that was recently accepted for publication in *Microsystems & Nanoengineering*. It provides a non-specialist audience with a synopsis of your key research outcomes and conclusions. This value-added service provided by Springer Nature is designed to raise interest in your research across the broader community.

Springer Nature will publish the summary on the journal’s website, and it will be freely available under a under the CC BY licence (Creative Commons Attribution v4.0 International Licence) (see the journal website for details). We encourage you to re-use the summary to bring attention to your research; for example, you can host it on your own website and share it via social-networking platforms. Please attribute the summary to *Microsystems & Nanoengineering* and your article (e.g. by providing a link to your article) and do not make derivatives.

Please note that to maximise the usefulness of these summaries they must follow several stringent guidelines:
-- Spelling, punctuation and style are set according to *Nature* editorial guidelines. As this summary is aimed at non-expert readers, some concepts and technical terms will be simplified.
-- Total length must be no more than 135 words. It is likely that not all points in the paper will be covered.
-- The first sentence must be no more than 280 characters, including spaces, to allow use on microblogging sites.
-- The headline must consist of a brief generic subject identifier followed by a short description. No more than 10 words in total.

Please contact the editorial office ([mems_nano@mail.ie.ac.cn](mailto:mems_nano@mail.ie.ac.cn)) immediately with corrections should you find any factual errors in this Editorial Summary.
